# Mortality among internal and international migrants in the 100 Million Brazilian Cohort

**DOI:** 10.1093/eurpub/ckac129.653

**Published:** 2022-10-25

**Authors:** JM Pescarini, E Goes, P Scaff, B Schindler, LC Rodrigues, EB Brickley, L Smeeth, ML Barreto

**Affiliations:** 1 Centre of Data and Knowledge Integration for Health, Oswaldo Cruz Foundation, Salvador, Brazil; 2 Faculty of Epidemiology and Population, LSHTM, London, Brazil

## Abstract

**Background:**

There is limited evidence on the health of migrant populations in low and middle-income countries (LMICs). Here, we investigated the patterns of mortality risk in migrants and non-migrants in women and men over the life course.

**Methods:**

We linked socioeconomic and mortality data from 1st Jan 2011 to 31st Dec 2018 in the 100 Million Brazilian Cohort. We calculated all-cause and cause-specific age-standardised mortality rates according to individuals’ migration status. Using Cox regression models, we estimated the age- and sex-adjusted mortality hazard ratios (HR) for internal migrants (i.e., people born in Brazil but living in a different Brazilian state to their state of birth) compared to Brazilian-born non-migrants; and for international migrants (i.e., people born in another country) compared to Brazilian-born individuals.

**Results:**

We followed 45,051,476 individuals, of whom 6,057,814 were internal migrants and 277,230 were international migrants. Internal migrants had a similar overall risk of all-cause mortality compared to Brazilian non-migrants (aHR=0.99, 95%CI=0.98-0.99), with lower mortality from some causes but higher mortality for some non-communicable diseases (NCDs). Compared to Brazilian-born individuals, international migrants had a lower risk of all-cause mortality (aHR=0.82, 95%CI=0.80-0.84), with up to 50% lower risk of death attributed to interpersonal violence among international migrant men (aHR=0.50, 95%CI=0.40-0.64), but a markedly higher risk of death by avoidable causes related to maternal health among young migrant women (aHR=2.17, 95%CI=1.17-4.05).

**Conclusions:**

Overall, internal migration was not associated with excess all-cause mortality, while international migration into Brazil was associated with lower all-cause mortality. Mortality patterns among migrant populations in Brazil show marked variation for specific causes of death, and risks varied by age and sex.

**Key messages:**

• Non-communicable diseases and maternal mortality are disproportionally higher among internal and international migrants, respectively.

• Further investigation of the underlying factors associated with higher maternal mortality among international migrant women is key to informing the targeting of social and health interventions.

